# Return to sport following navicular stress fracture: a systematic review and meta-analysis of three hundred and fifteen fractures

**DOI:** 10.1007/s00264-021-05147-6

**Published:** 2021-08-20

**Authors:** Ahmed Khalil Attia, Karim Mahmoud, Jason Bariteau, Sameh A. Labib, Christopher W. DiGiovanni, Pieter D’Hooghe

**Affiliations:** 1 Penn State Milton S. Hershey Medical Center, 500 University Drive, Hershey, PA USA; 2 Emory University Hospital, Atlanta, GA USA; 3 Harvard Medical School, Boston, MA USA; 4 Aspetar Orthopaedic and Sports Medicine Hospital, Doha, Qatar

**Keywords:** Navicular stress fracture, Tarsal navicular, Athletes, Return to play, Operative management, Conservative management

## Abstract

**Purpose:**

This meta-analysis aims to provide updated evidence on the success rate, return to play (RTP) rate, time to RTP, and complications of operatively and conservatively managed navicular stress fractures (NSFs) as well as delays in diagnosis while avoiding limitations of previous similar studies.

**Methods:**

Following the Preferred Reporting Items for Systematic Reviews and Meta-Analyses (PRISMA) guidelines, two independent team members electronically searched MEDLINE (PubMed), EMBASE, Google Scholar, SCOPUS, and Cochrane databases throughout February 2021 using the following keywords with their synonyms: “Navicular stress fracture,” “return to play,” and “athletes.” The primary outcomes were (1) management success rate, (2) RTP rate, and (3) time to RTP. The secondary outcomes were (1) non-union, (2) time to diagnosis, (3) refracture, and (4) other complications. Inclusion criteria were clinical studies on NSFs reporting at least one of the desirable outcomes. Studies not reporting any of the outcomes of interest or the full text was not available in English, German, French, or Arabic were excluded. Case reports, case series with less than ten cases, and studies reporting exclusively on navicular non-union management were also excluded. The Newcastle–Ottawa scale was used for quality assessment while Review Manager (RevMan) Version 5.4 was used for the risk of bias assessment. Data were presented by type of treatment (surgical or conservative). If enough studies were present that were clinically and statistically homogeneous and data on them adequately reported, a meta-analysis was performed using a fixed-effects model. In case of statistical heterogeneity, a random-effects model was used. If meta-analysis was not possible, results were reported in a descriptive fashion. The need to explore for statistical heterogeneity was determined by an *I*^2^ greater than 40%.

**Results:**

Eleven studies met the inclusion criteria with a total of 315 NSF. Out of those, 307 (97.46%) NSFs were in athletes. One hundred eight (34.29%) NSFs were managed operatively, while 207 (65.71%) NSFs were managed conservatively. Successful outcomes were reported in 104/108 (96.30%) NSF treated operatively with a mean success rate of 97.9% (CI: 95.4–100%, *I*^2^ = 0%). Successful outcomes were reported in 149/207 (71.98%) NSF treated conservatively, with a mean success rate of 78.1% (CI: 66.6–89.6%, *I*^2^ = 84.93%). Successful outcome differences were found to be significant in favor of operative management (OR = 5.52, CI: 1.74–17.48, *p* = 0.004, *I*^2^ = 4.6%). RTP was noted in 97/98 (98.98%) NSF treated operatively and in 152/207 (73.43%) NSF treated conservatively, with no significant difference between operative and conservative management (OR = 2.789, CI: 0.80–9.67, *p* = 0.142, *I*^2^ = 0%). The pooled mean time to RTP in NSF treated operatively was 4.17 months (CI: 3.06–5.28, *I*^2^ = 92.88%), while NSF treated conservatively returned to play at 4.67 months (CI: 0.97–8.37, *I*^2^ = 99.46%) postoperatively, with no significant difference between operative and conservative management (SMD =  − 0.397, CI: − 1.869–1.075, *p* = 0.60, *I*^2^ = 92.24). The pooled mean duration of symptoms before diagnosis was 9.862 (3.3–123.6) months (CI: 6.45–13.28, *I*^2^ = 94.92%), reported in ten studies. Twenty (23.53%) refractures were reported after conservative management of 85 NSFs, while one (1.28%) refracture was reported after operative management of 78 NSFs, with a significant difference in favor of operative management (OR = 0.083, CI: 0.007–0.973, *p* = 0.047, *I*^2^ = 38.78%).

**Conclusion:**

Operative management of NSF provides a higher success rate, a lower refracture rate, and a lower non-union rate as compared to other non-operative management options. While not significant, there is a notable trend towards superior RTP rates and time to RTP following operative management. Therefore, we recommend operative fixation for all NSFs type I through III in athletes. Athletes continue to exhibit an alarmingly long duration of symptoms before diagnosis is made; a high index of suspicion must be maintained, therefore, and adjunct CT imaging is strongly recommended in the case of any work-up. Unfortunately, the published literature on NSFs remains of lower level of evidence and high-quality studies are needed.

## Introduction

Formal recognition of the navicular stress fracture (NSF) has been relatively recent, with its first description by Towne et al. in 1970 [1]. While rare injuries in the general population, NSFs are becoming increasingly recognized in athletes, with an estimated prevalence comprising up to 35% of stress fractures in the foot and ankle [2]. This can be attributed to the increased awareness of the injury, paired with the adoption of advanced imaging modalities which had significantly improved the diagnostic accuracy of NSF [3]. In fact, the most widely used classification of NSF by Saxena et al. classifies NSF based on fracture morphology on CT scans into types 1 to 3 [4]. Type 0.5 was later added based on MRI findings [5] (Table 1).

**Table 1:** Saxena et al. navicular stress fracture classification^a^

Type		Findings
0.5	MRI	Stress reaction and bone edema. Normal CT
1	CT	Unicortical fracture: dorsal only
2	CT	Dorsal fracture propagating into the body
3	CT	Bicortical fracture: dorsal + plantar, medial, or lateral
**Modifiers**		
A	Avascular necrosis	
C	Cystic degeneration	
S	Sclerosis of fracture lines	

^a^Adapted from Saxena et al.[4, 5]

NSFs commonly affect athletes who participate in running and repetitive high impact sports such as track and field and basketball. Several factors have been suggested to predispose the navicular bone to stress injury. Forces transmitted from the first and second TMT joints pass through the navicular unto the talar head medially, while the lateral aspect of the navicular does not share in this force transmission. Moreover, the contraction of the tibialis posterior attached to the medial aspect of the navicular creates tension forces medially [3, 6]. This creates significant shear forces across the middle third of the navicular bone which is most vulnerable area due to its watershed hypo-vascularity [3, 7]. The repetitive and high stresses of intense athletic activities create microfractures that have limited healing potential. Other factors such as female sex [8], decreased plantar flexion and forefoot abduction, and greater hindfoot valgus have also been linked to NSF [9].

The optimal management of NSFs has been a matter of debate for the last few decades. Earlier literature suggested that conservative management in the form of non-weight-bearing had a comparable success rate with surgical management [10]. However, this view is challenged by more recent studies recommending surgical management, reflecting a more aggressive management strategy of NSFs in athletes [3].

This meta-analysis is not the first study to report on the management of NSFs. A landmark meta-analysis was performed by Torg et al. in 2010 [10]. Since then, three studies adding another 87 NSFs have been published in the literature, with a relatively higher level of evidence [11–13]. Most other reports to date have been systematic reviews summarizing case reports and small case series [3, 10, 14, 15].

## Purpose

This meta-analysis aims to provide updated evidence on the success rate, return to play (RTP) rate, time to RTP, and complications of operatively and conservatively managed NSF as well as delays in diagnosis while avoiding limitations of previous similar studies.

## Materials and methods

The current meta-analysis was performed according to the Preferred Reporting Items for Systematic Reviews and Meta-analyses (PRISMA) guidelines [16].

### Literature search

Relevant studies were identified from database inception to February 2021. Electronic-based search on MEDLINE (PubMed), EMBASE, Google Scholar, SCOPUS, and Cochrane databases using the following keywords with their synonyms and combinations of these keywords: “Navicular stress fracture,” “return to play,” and “athletes.” In addition, the reference lists from previous review articles were searched manually to check for eligible studies. Additionally, abstracts of articles published in *American Journal of Sports Medicine*, *Orthopaedic Journal of Sports Medicine*, *Foot and Ankle International*, *Foot and Ankle Surgery*, *Journal of Foot and Ankle Surgery*, *Foot and Ankle Specialist*, and *Foot and Ankle Orthopaedics Journals* were manually searched for relevant articles.

Two investigators (XX, XX) independently reviewed all titles, abstracts, and the full text of potentially eligible articles based on the abstract review. Full texts in German were reviewed by one investigator (XX). The eligible studies were selected according to the inclusion and exclusion criteria detailed below. Any disagreement was resolved by discussion to reach a unanimous decision. Any further conflict was resolved by the senior authors (XX, XX).

### Study eligibility criteria

The research team systematically reviewed published studies according to the following inclusion criteria: clinical studies on NSFs reporting at least one of the desirable outcomes (RTP rate, time to RTP, success rate, time to diagnosis, non-union rate, or refracture rate). Exclusion criteria were: studies not reporting any of the outcomes of interest or the full text was not available in English, German, French, or Arabic; case reports, case series with less than 10 cases, and studies reporting exclusively on navicular non-union management.

The primary outcomes were: (1) management success rate, (2) RTP rate, and (3) time to RTP. The secondary outcomes were (1) non-union, (2) time to diagnosis, (3) refracture, and (4) other complications. We adopted the definition of successful outcome described by Torg et al., “an outcome in which the patient was pain-free, able to return to previous activity level, and did not have recurrence of the fracture” [10].

### Data collection

The data retrieved included the following: study characteristics (title, authors, year, level of evidence), subjects’ characteristics (age, gender, follow-up, level of athletic activity, and the type of sport), management characteristics, and the outcomes measures.

### Data synthesis and analysis

Data were presented by type of treatment (surgical or conservative). If enough studies were present that were clinically and statistically homogeneous and data on them adequately reported, a meta-analysis was performed using a fixed-effects model. In case of statistical heterogeneity, a random-effects model was used. If meta-analysis was not possible, results were reported in a descriptive fashion. The need to explore for statistical heterogeneity was determined by an *I*^2^ greater than 40%.

Statistical analysis was carried out by the first author (XX) and reviewed by an independent statistician. The data analysis was done by Review Manager (RevMan) Version 5.4, The Cochrane Collaboration, 2020 [Computer program] using a random-effect model, Comprehensive Meta-analysis Software (Biostat Inc, Englewood, NJ, USA), and SPSS 25 (IBM Corp, Armonk, NY, USA). The standardized mean difference (SMD) and 95% confidence interval (CI) were calculated for continuous variables. For nominal variables, odds ratio (OR) and 95% confidence interval (CI) were calculated. Values of *p* < 0.05 were considered statistically significant. Heterogeneity was assessed using Higgins-*I*^2^ methods. Ranges for interpretation of *I*^2^, according to the Cochrane Handbook for Systematic Reviews of Interventions, were 0–40% (poor), 30–60% (fair), 50–90% (moderate), and 75–100% (considerable) [17].

### Risk of bias assessment

**Fig. 1:**
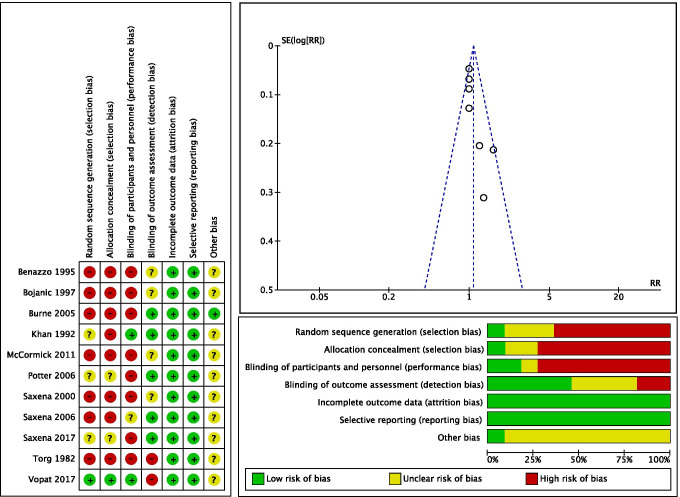
Funnel plot of the return to play rate and risk of bias assessment summary in included studies

The Newcastle–Ottawa Quality Assessment scale [18] was used for the quality assessment by two independent investigators. Newcastle–Ottawa scale looks for the quality of the study from three domains: Selection, Comparability, and Outcome/Exposure (Table 2). Additionally, Review Manager (RevMan) Version 5.4, The Cochrane Collaboration, 2020 [Computer program] was used for the risk of bias assessment. To assess the risk of publication bias, a funnel plot of the most reported outcome (RTP rate) was charted. The plot detected good symmetrical distribution of the referral points. Almost all the values are narrow to the no-effect line and none outside the range of acceptability. It showed poor data dispersion, confirming a low risk of publication bias of the current study (Fig. 1). The level of evidence was assigned according to the Cochrane Book Review Group [17].

**Table 2:** Quality assessment of included studies according to the Newcastle–Ottawa scale [18]

No	Study	Yr	LOE	Selection	Comparability	Outcome/exposure
1	Vopat et al. [12]	2017	III	***	_	***
2	Saxena et al. [11]	2017	II	****	*	***
3	McCormick et al. [13]	2011	IV	***	_	***
4	Saxena and Fullem [19]	2006	III	****	*	***
5	Potter et al. [20]	2006	IV	****	*	***
6	Burne et al. [21]	2005	IV	***	_	***
7	Saxena et al. [4]	2000	IV	****	*	***
8	Bojanic et al. [22]	1997	IV	***	_	***
9	Benazzo et al. [23]	1995	IV	***	_	**
10	Khan et al. [24]	1992	IV	***	_	***
11	Torg et al. [25]	1982	IV	***	_	**

The number of * reflects the number of points achieved on each domain

## Results

**Fig. 2:**
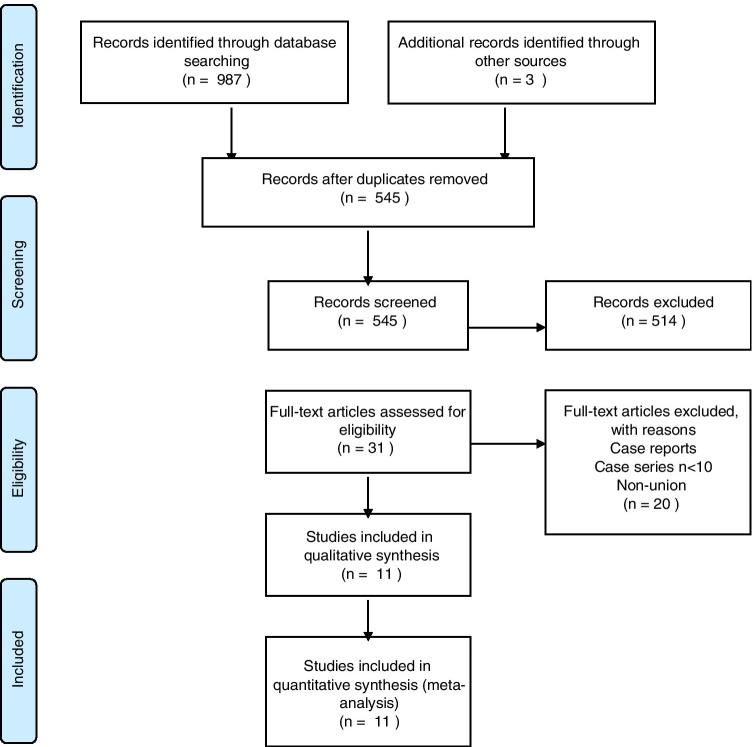
PRISMA flowchart

Following removal of duplicate entries, a total of 545 studies were identified. Upon careful screening of these remaining records, 31 studies met criteria for full-text assessment. Eleven studies qualified for the meta-analysis [4, 11–13, 19–25] (Fig. 2, Table 3). A total of 315 NSF in 307 patients were included. Out of 293 patients whose gender was reported, 147 (50.17%) were males, while 146 (49.83%) were females. Three hundred seven (97.46%) NSFs were in athletes. The mean age was 24.58 years (CI: 22.01–27.16, *I*^2^ = 89.5). The mean follow-up length was 45.77 months (95%CI: 34.44–57.09, *I*^2^ = 95.69%). Out of 315 NSFs, 108 (34.29%) were managed operatively, while 207 (65.71%) were managed conservatively.

**Table 3:** Characteristics of included studies

No	Study	Yr	Country	NSF (*N*)	Age ^a^ (Yr)	Symptoms duration^a^ (mon)	Athletes	Main sport	Intervention	N	RTP rate	Mean time to RTP^a^ (mon)	Success of management
1	Vopat et al	2017	USA	15	NR	22	14/14	Football	Operative	9	9 (100%)	NR	8/9
									Conservative	6	6 (100%)	NR	5/6
2	Saxena et al	2017	USA	62	29.6	8.8	62/62	Track and field	Operative	47	47 (100%)	5.56	47/47
									Conservative	15	15 (100%)	3.97	7/15
3	McCormick et al	2011	USA	10	28.7	4.25	7/10	Football	Operative	10	NR	NR	8/10
4	Saxena and Fullem	2006	USA	19	24.7	18.18	19/19	NR	Operative	13	13 (100%)	4.1	13/13
									Conservative	6	5 (83.3%)	3.7	5/6
5	Potter et al	2006	Australia	29	33.5	123.6	26/26	NR	Operative	12	12 (100%)	NR	12/12
									Conservative	17	17 (100%)	NR	17/17
6	Burne et al	2005	Canada	20	22.5	3.75	20/20	NR	Conservative	20	12 (60%)	NR	14/20
7	Saxena et al	2000	USA	22	27.2	5.04	22/22	Track and field	Operative	9	9 (100%)	3.11	9/9
									Conservative	13	13 (100%)	4.35	8/13
8	Bojanic et al	1997	France	18	20.1	3.3	17/17	Track and field	Conservative	18	18 (100%)	2	18/18
9	Benazzo et al	1995	Italy	13	21.8	7.97	13/13	Track and field	Conservative	13	13 (100%)	NR	13/13
10	Khan et al	1992	Australia	86	20.20	NR	82/82	NR	Operative	6	5 (83.3%)	3.8	5/6
									Conservative	80	41 (51.3%)	9.3	50/80
11	Torg et al	1982	USA	21	21.8	7.2	NR	Track and field	Operative	2	2/2	NR	2/2
									Conservative	19	12/19	NR	12/19

*Abbreviations*: *Yr* year, *NSF* navicular stress fractures, *N* number, *Mon* month(s), *RTP* return to play, *NR* not reported

^a^Data expressed as means

The level of athletic participation was reported for 236 (78.87%) out of the 307 athletes included in the study [4, 11–13, 19–24]. Of those 236 athletes, 73 (30.93%) were elite, 26 (11.02%) were collegiate, and 108 (45.76%) were recreational athletes. Sixteen (6.78%) were described as “competitive athletes” [19], while 13 (5.51%) were described as “regional” [23]. The type of sport played was reported for 155 athletes (95.77%) [4, 11, 12, 22–25]. Of those 155 athletes, 81 (52.26%) were involved in running activities, including track and field, 21 (13.55%) in football, 11 (7.10%) in basketball, and 40 (27.09%) in other sports.

### Success rate

**Fig. 3:**
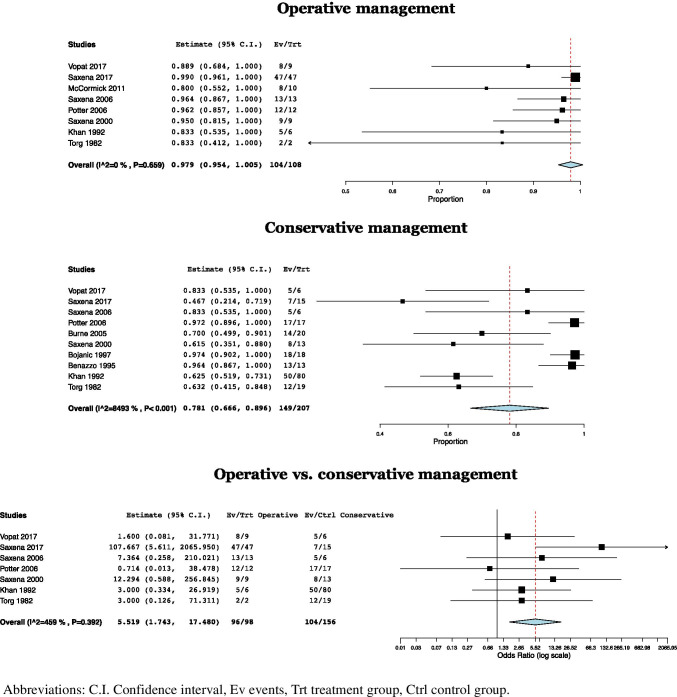
Forest plots of success rates in operative management, conservative management, and operative vs. conservative management of navicular stress fractures. Abbreviations: C.I., confidence interval; Ev, events; Trt, treatment group; Ctrl, control group

Successful outcomes were reported in 104/108 (96.30%) NSF treated operatively with a mean success rate of 97.9% (CI: 95.4–100%, *I*^2^ = 0%) [4, 11–13, 19, 20, 24, 25]. Successful outcomes were reported in 149/207 (71.98%) NSF treated conservatively, with a mean success rate of 78.1% (CI: 66.6–89.6%, *I*^2^ = 84.93%) [4, 11, 12, 19–25]. Outcome differences were found to be significant and in favor of operative management (OR = 5.52, CI: 1.74–17.48, *p* = 0.004, *I*^2^ = 4.6%) [4, 11, 12, 19, 20, 24, 25] (Fig. 3).

### Duration of symptoms

**Fig. 4:**
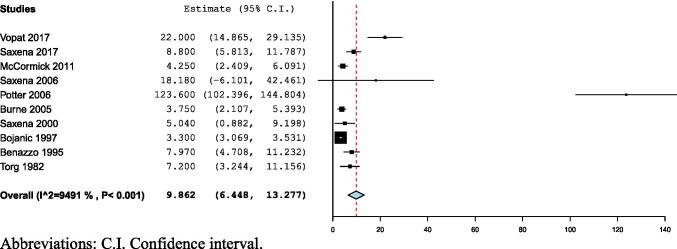
Forest plot of duration of symptoms before diagnosis of NSF. Abbreviations: C.I., confidence interval

The pooled mean duration of symptoms before diagnosis was 9.862 (3.3–123.6) months (CI: 6.45–13.28, *I*^2^ = 94.92%), reported in ten studies [4, 11–13, 19–23, 25] (Fig. 4).

### Return to play

**Fig. 5:**
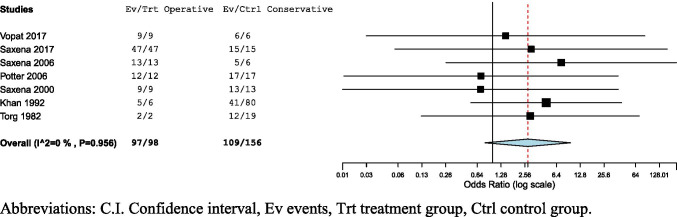
Return to play rate after operative versus conservative management of navicular stress fractures. Abbreviations: C.I., confidence interval; Ev, events; Trt, treatment group; Ctrl, control group

The overall RTP rate was found to be 249 (81.64%) out of 305 NSF, reported across 10 studies (4, 11–12, 19–25). RTP was noted in 97/98 (98.98%) NSF treated operatively and in 152/207 (73.43%) NSF treated conservatively. There was no significant difference found in RTP rates following operative versus conservative management (OR = 2.789, CI: 0.80–9.67, *p* = 0.142, *I*^2^ = 0%) [4, 11–12, 19–20, 24–25) (Fig. 5).

**Fig. 6:**
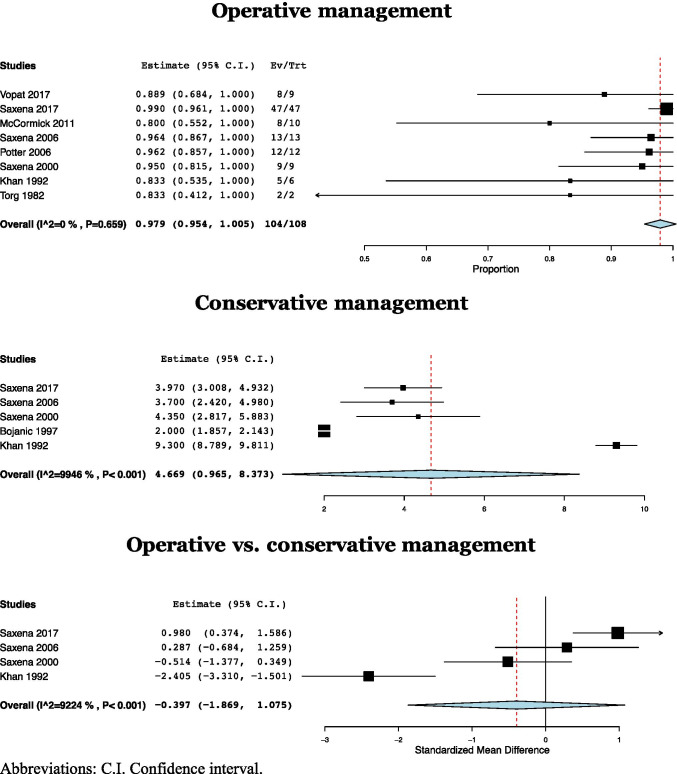
Time to return to play rate after operative, conservative, and operative versus conservative management of navicular stress fractures. Abbreviations: C.I., confidence interval

The pooled mean time to RTP in NSF treated operatively was 4.17 months (CI: 3.06–5.28, *I*^2^ = 92.88%) [4, 11, 19, 24], while NSF treated conservatively returned to play at 4.67 months (CI: 0.97–8.37, *I*^2^ = 99.46%) postoperatively [4, 11, 19, 22, 24]. There was no significant difference in time to RTP between operatively and conservatively managed NSF (SMD =  − 0.397, CI: − 1.869–1.075, *p* = 0.60, *I*^2^ = 92.24%) [4, 11, 19, 24] (Fig. 6).

### Refracture

Refracture rate was reported in seven studies [4, 11, 12, 19, 21–23]. In total, 21 (12.88%) NSFs were complicated by refracture. Twenty (23.53%) refractures were reported after conservative management of 85 NSFs [4, 11, 12, 19, 21–23], while one (1.28%) refracture was reported after operative management of 78 NSFs [4, 11, 12, 19], a significant difference (OR = 0.083, CI: 0.007–0.973, *p* = 0.047, *I*^2^ = 38.78%).

### Non-union

Non-union rate was reported in eight studies [4, 11–13, 19, 21, 23, 24]. In total, there were 36 non-unions representing 14.57% of NSFs. Out of 94 operatively treated NSFs, there were three (3.19%) non-unions [4, 11–13, 19, 24], in comparison to 33 (21.57%) non-unions out of 153 conservatively treated NSFs [4, 11, 12, 19, 21, 23, 24].

## Discussion

To this day, NSF continues to be a diagnostic challenge often complicated by substantial delay in diagnosis. Meta-analysis of reportedly symptomatic athletes suggests that it takes on average over nine months to make the diagnosis of NSF. In one study, it took up to ten years to make the correct diagnosis and provide treatment [20]. Saxena and Fullem also noted a relationship between delayed RTP and longer duration of symptoms, suggesting that duration of symptoms correlated with advancing fracture severity [19]. Left untreated, these fractures can propagate and lead to progressive degenerative change of the talonavicular joint, further complicating both management and outcome. Numerous factors contribute to this diagnostic challenge. First, as with many overuse syndromes, presentation can be insidious; patients often describe only vague pain with weight-bearing and sports-specific medial foot pain. Moreover, examination can be unremarkable, except for occasional tenderness to palpation over the most dorsal aspect of the talonavicular joint, *the N-spot* [24], rarely accompanied by subtle bruising and swelling due because of an inherently poor blood supply [3, 10, 11, 15]. Further, plain radiographs have limited sensitivity because these fractures are often nondisplaced or incomplete and because substantial resorption is required before any fracture plane becomes visible [3, 11]. It should also be noted that the complex three dimensional shape of the navicular overlaps other bones and makes diagnosis even more challenging. Khan et al. reported the plain radiographs were positive for NSF in only 14 out of 77 CT confirmed NSFs, a modest sensitivity of only 18% [24]. Hence, advanced imaging modalities have been recommended. Bone scans have been reported to approach 100% sensitivity but unfortunately lack specificity and have limited utility for discerning fracture morphology or displacement [26]. CT scans and MRIs have therefore become more widely recommended, with many authors now considering CT scan to be the gold standard, with up to 100% accuracy [11, 25, 27]. The prolonged delay in diagnosis despite the accuracy of CT scans might reflect a high threshold for ordering advanced imaging modalities at initial presentation. Regardless of the chosen imaging modality, a high index of suspicion and a low threshold for CT scan and MRI scans if CT scans are inconclusive are strongly advised when managing athletes who present with N-spot tenderness or simply localize complaints to this region despite normal plain radiographs and an otherwise vague clinical presentation [3].

NSFs can have serious consequences on any athletic career, and this needs to be discussed with both the players and their coaches. We found that it takes an athlete, on average, more than four months to RTP. This means they can miss up to one full season if delay in diagnosis is also factored in. In a study on the National Football League (NFL) Combine by Vopat et al., football players with NSF were more likely to have ipsilateral talonavicular arthritic changes in comparison to the uninjured side [12]. Moreover, those players were less likely to be drafted for the NFL, and if they were drafted, they played fewer games and were less than half as likely to continue playing for more than two years in comparison to players with no NSFs [12].

Optimal management for NSF remains a matter of debate. Our data suggest that management was 5.5 times more likely to be successful following operative as compared to conservative management, although it is still unclear if these two populations presented with what could be considered equivalent and comparable initial fracture patterns before management ensued. Nonetheless, operative management in our population exhibited lower rates of refracture and non-union than did those managed conservatively. Notably, all but one refracture included in these studies occurred after conservative management [12]. There was a trend towards the superiority of the operative management over conservative management in terms of RTP rates (99% vs. 73%, respectively) and time to RTP (4.2 vs. 4.7 months, respectively). However, this difference did not reach significance. Interestingly, our findings contradict those of Torg et al. in their landmark meta-analysis—where they concluded that conservative management had superior outcomes to operative management [10]. It appears that higher level evidence studies will be required to reach more definitive conclusion about optimal management. At the moment, however, we strongly support the recommendations by Saxena et al. and Patel et al. of operative management of all Saxena type II and III NSFs in athletes [3, 5, 11]. Strong consideration should be given to operative management of type I NSF in elite athletes to avoid complications. Conservative management, in the form of strict non-weight-bearing in a boot for six to eight weeks, should be reserved for undisplaced fractures in recreational athletes and only type 0.5 in elite athletes. Regardless of the management and fracture type, athletes should be kept non-weight-bearing and must not be cleared for RTP until evidence of fracture union is confirmed on CT scans.

Multiple operative techniques have been described based on the fracture classification, displacement, location, and surgeon’s preference. In undisplaced Saxena type I fractures, bone grafting can be sufficient although compression screw fixation is recommended to reduce the risk of displacement [3]. For displaced fractures, open reduction through a medial approach is most commonly used, but a lateral incision can be used to tackle lateral fractures and aid screw positioning [3]. Patel et al. recommended adding iliac crest bone marrow aspirate concentrate and cancellous bone autografts to augment the fracture fixation [3]. They recommended using one or two partially threaded 3.5 mm cannulated lag screws placed from medial to lateral under 3D radiographic imaging. Saxena et al. used a dorsal incision lateral to the neurovascular bundle and extended the dissection to the talonavicular joint [11]. They used a 4.0 mm partially threaded solid cancellous screw placed from dorsally and laterally to plantarly and medially toward the navicular tuberosity. The fixation was augmented by calcaneal cancellous autografts and occasionally with platelet-rich plasma (PRP) [11].

### Limitations

Despite the authors’ best efforts, this study is not without limitations. Similar to the drawbacks of all meta-analyses, our data pool is subject to the limitations of population heterogeneity, and the unknown bias in primary studies. The major limitation of the current study is the overall small number of subjects included due to the rarity of NSF. That said, however, it should also be noted that this study represents the largest study to date that excludes case reports and small case series when performing data analysis and basing its conclusions.

We are unable to explain why the improved success rates and lower refracture and non-union rates did not result in improved RTP parameters. It is possible that our study was underpowered to detect the difference. Furthermore, RTP might also be influenced by other variables such as the athlete’s desire for a quick return, the talent of the individual, scholarship or contract implications, presence of other injuries, career length, and timing of the injury in relation to the sports season [28].

Additionally, the level of evidence of included studies was generally low. Eight studies out of 11 were level IV, while two were level III and only one study was level II. This low level of evidence in combination with the considerable heterogeneity in some of the outcome measures precludes making unequivocal conclusions and high-quality studies are still required. However, this meta-analysis represents the best available evidence to date.

We also recognize the potential bias when comparing operative and conservative management. Some of the authors used bone grafts in operative management, while some used electrical stimulation in conservative management. Moreover, some NSFs were allowed weight-bearing, and others were non-weight-bearing for different durations. Also, the decision to treat an injury operatively or conservatively might be a reflection of its severity, geographical differences in management protocols, and patients’ preferences.

## Conclusion

Operative management of NSF provides a higher success rate, a lower refracture rate, and a lower non-union rate as compared to other non-operative management options. While not significant, there is a notable trend towards superior RTP rates and time to RTP following operative management. Therefore, we recommend operative fixation for all NSFs type I through III in athletes. Athletes continue to exhibit an alarmingly long duration of symptoms before diagnosis is made; a high index of suspicion must be maintained, therefore, and adjunct CT imaging is strongly recommended in the case of any work-up. Unfortunately, the published literature on NSFs remains of lower level of evidence and high-quality studies are needed.

## Data Availability

Not applicable.
